# Use of biological based therapy in patients with cardiovascular diseases in a university-hospital in New York City

**DOI:** 10.1186/1472-6882-5-4

**Published:** 2005-03-03

**Authors:** Larisa Chagan, Diane Bernstein, Judy WM Cheng, Harold L Kirschenbaum, Vitalina Rozenfeld, Gina C Caliendo, Joanne Meyer, Bernard Mehl

**Affiliations:** 1 Division of Pharmacy Practice, Arnold & Marie Schwartz College of Pharmacy and Health Sciences, Long Island University, Brooklyn, New York, USA; 2 At the time of the study, Arnold & Marie Schwartz College of Pharmacy and Health Sciences, Long Island University, Brooklyn, New York, USA; 3 Medical Science Liaison, Watson Laboratories, Inc., Morristown, New Jersey, USA; 4 Department of Pharmacy, Mount Sinai Hospital, New York, New York, USA

## Abstract

**Background:**

The use of complementary and alternative products including Biological Based Therapy (BBT) has increased among patients with various medical illnesses and conditions. The studies assessing the prevalence of BBT use among patients with cardiovascular diseases are limited. Therefore, an evaluation of BBT in this patient population would be beneficial. This was a survey designed to determine the effects of demographics on the use of Biological Based Therapy (BBT) in patients with cardiovascular diseases. The objective of this study was to determine the effect of the education level on the use of BBT in cardiovascular patients. This survey also assessed the perceptions of users regarding the safety/efficacy of BBT, types of BBT used and potential BBT-drug interactions.

**Method:**

The survey instrument was designed to assess the findings. Patients were interviewed from February 2001 to December 2002. 198 inpatients with cardiovascular diseases (94 BBT users and 104 non-users) in a university hospital were included in the study.

**Results:**

Users had a significantly higher level of education than non-users (college graduate: 28 [30%] versus 12 [12%], p = 0.003). Top 10 BBT products used were vitamin E [41(43.6%)], vitamin C [30(31.9%)], multivitamins [24(25.5%)], calcium [19(20.2%)], vitamin B complex [17(18.1%)], fish oil [12(12.8%)], coenzyme Q10 [11(11.7%)], glucosamine [10(10.6%)], magnesium [8(8.5%)] and vitamin D [6(6.4%)]. Sixty percent of users' physicians knew of the BBT use. Compared to non-users, users believed BBT to be safer (p < 0.001) and more effective (p < 0.001) than prescription drugs. Forty-two potential drug-BBT interactions were identified.

**Conclusion:**

Incidence of use of BBT in cardiovascular patients is high (47.5%), as is the risk of potential drug interaction. Health care providers need to monitor BBT use in patients with cardiovascular diseases.

## Background

The use of complementary and alternative medicine (CAM), defined by the United States National Center for CAM as a group of diverse medical and health care systems, practices, and products that are not presently considered to be a part of conventional medicine, has grown tremendously in the United States [1-4]. A recent national survey reported that four out of every ten Americans used at least one form of CAM, and one out of five used prescription medications together with CAM [3]. The prevalence is even higher in patients with chronic medical problems (for example, 28 to 90% in patients with arthritis, 11–56% in those with cancer, 60% in patients with asthma and 67.8% in patients with human immunodeficiency virus) [5-11]. Biological based therapies (BBT) is an important type of CAM and is defined by the National Center for CAM as use of substances found in nature, such as herbs, foods, and vitamins. BBT is the second most commonly utilized CAM, with the first being prayer therapy [2].

Similar to other chronic medical conditions, patients suffering from a variety of cardiovascular diseases including coronary heart disease, congestive heart failure, stroke, arrhythmia and congenital cardiovascular defects, may also be looking to CAM to prevent or treat their illnesses. This is particularly likely since a number of BBT products including vitamin E, vitamin C, beta-carotene, fish oils, and coenzyme Q10 have been evaluated for prevention and/or treatment of cardiovascular diseases [12-25].

Despite a wide array of available BBT for cardiovascular conditions, studies evaluating the prevalence of usage of these agents are limited [21-25]. As CAM, in general, has become widely accessible to the public, and BBT may be purchased in pharmacies, health food stores and supermarkets, it is difficult to control patient usage of these products. In addition, the likelihood for adverse effects and interactions between conventional therapies and BBT places patients using such products at an increased risk of adverse drug events. It is, therefore, important to examine patient usage so as to advise and monitor them properly. Among the studies conducted to-date that included patients with a broad spectrum of cardiovascular diseases, few focused only on BBT. The studies examined different factors that may determine BBT use, but none of them examined the potential for side effects and drug interactions with other prescription and non-prescription medications that the patients were utilizing. Since patients with cardiovascular conditions consume many prescription medications with narrow therapeutic indexes and extensive drug interaction profiles [26,27], it is important to look at the prevalence of use and potential drug interactions in a cohort of cardiovascular patients.

The primary objective of this study was to determine the effect of the education level on the use of BBT in cardiovascular patients. The study also investigated the attitudes and beliefs towards BBT by patients with cardiovascular diseases. In addition, patient perceptions regarding the safety and efficacy of BBT, common BBT used and a list of potential BBT-drug interactions were reviewed.

## Methods

This is a cross-sectional, descriptive study utilizing structured interviews to assess the level of education, usage, beliefs and attitudes towards BBT among inpatients with cardiovascular diseases. The study was conducted in the Cardiac Care Center at the Mount Sinai Hospital, New York, USA from February 2001 to December 2002. Participants provided informed consent and were interviewed by one of the investigators. To maintain consistency of the interview and to prevent interviewer bias, a scripted letter was drafted for the investigators to invite the patients to participate in the study and to explain the process of the study. After a patient was enrolled, an investigator read the survey questions verbatim to the patient and tried not to elaborate whenever possible. Patients were included in the study if they had at least one of the following diagnoses: cardiovascular disease(s) including congestive heart failure, coronary heart disease, thromboembolic diseases, valvular heart disease, arrhythmia, vascular aneurysm, peripheral vascular disease, pulmonary hypertension, congenital heart disease and post heart transplant. Additional inclusion criteria included being 18 years of age or older, English speaking, no documented cognitive deficits precluding the patient from understanding the interviewer, and willingness to provide an informed consent. Prior to patient contact, the attending physicians of the eligible patients were contacted and informed about the study. If the attending physicians chose not to have their patients participate in the study, the patients were not included. Upon agreement of the physicians, subjects were invited to participate and were asked to sign an informed consent at their convenience prior to being interviewed. This study was approved by the Institutional Review Boards of Mount Sinai Hospital and Long Island University.

### Biological based therapy survey

Utilizing a structured instrument (see Additional file 1), eligible subjects were interviewed by one of the investigators during their stay at the hospital. The BBT survey was modified and adapted from a previously published survey [6]. The participants could choose to answer or not to answer any question at their discretion, and could discontinue their participation in the study at any time during the interview. During the interview, demographic data, including age, gender, race, marital status, level of education, annual income, and working status, were collected. Additionally, history of cardiovascular and other medical conditions, and medications utilized were recorded. The definition of BBT in this study was similar to that defined by the United States National Center for CAM, which included all herbal supplements, vitamins and mineral supplements. The patients' attitudes and beliefs towards BBT were assessed by asking them about their perceived safety and efficacy of BBT. The side effects and potential drug/food interactions listed by the patients were compared against those listed in the MicroMedex^® ^Database [28]. The patients' assessments of benefits of BBT as compared with conventional medicine were recorded. Additionally, the participants were asked whether they reported the use of BBT to their physicians, pharmacists, or other healthcare professionals. A review of patients' medical records was performed to collect data about patients' cardiovascular diseases and to confirm medications used. Although, the identity of the participants in this research study was kept confidential, patients were notified in the informed consent process that if potential BBT-prescription medication interactions were identified, their physicians would be notified.

### Statistical analysis

For this study to have an 80 percent power to detect a 20 percent clinically significant difference in determining factors of BBT use such as education level between the users and non-users of BBT, and establishing a p value of < 0.05 as the level of statistical significance, approximately 200 patients (100 patients in each group) needed to be enrolled. For demographic parameters, continuous variables were compared between the two groups using Students' t-test and categorical variables were compared using chi-square. Attitudes and beliefs regarding the safety and efficacy of BBT were compared between the two groups using a chi-square test for categorical data and Students' t test for Likert-type scale questions. The BBT products used by cardiovascular patients were recorded and the likelihood of potential drug interactions between BBT and other medications the patients were taking were described. Statistical analyses were conducted using Statistical Product and Service Solutions program (SPSS^® ^for Windows, Rel. 10.01 1999).

## Results

A total of 200 patients who were admitted to the Cardiac Care Center at Mount Sinai Hospital were enrolled into the study. All the physicians approached agreed to have their patients participate in the survey. Two of the patients were, eventually, excluded from data analysis due to incomplete survey data. Of the remaining 198 patients, 94 (47.5%) reported BBT use at some point in their lifetime and 84 (42%) reported using such products during the immediate 12 months prior to the survey. Of the 94 patients who used BBT, 32% reported using the products all the time.

### Demographic characteristics

The demographic and socioeconomic characteristics of the total sample are presented in Table 1. The mean age of surveyed participants was 60.0 ± 16.5 years, and their ages were normally distributed ranging from 19 to 102 years of age. The overall sample consisted of 118 (59.6%) men and 80 (40.4%) women.

**Table 1:** Demographic Characteristics of Study Subjects

		**Users (%)**	**Nonusers (%)**	**p value**
**Total**^1^	N = 198	94 (47.5)	104(52.5)	
**Age**^2^		61.4 ± 16.7	58.7 ± 16.3	NSS
**Gender**				NSS
Male		51(54)	67(64)	
Female		43(46)	37(36)	
**Race**				NSS
White		53(56)	46(44)	
Black		19(20)	27(26)	
Hispanic		12(13)	18(17)	
Other^3^		10(11)	13(7)	
**Education**				0.0006
< High school		12(13)	28(27)	
High school		23(24)	42(40)	
Some college		18(19)	12(12)	
College Graduate		28(30)	12(12)	
Graduate School		13(14)	8(8)	
**Annual Household Income**				NSS
< $10,000		17(27)	19(30)	
$10,000–$30,000		12(19)	15(23)	
$30,000–$50,000		8(13)	14(22)	
$50,000–$75,000		15(23)	7(11)	
$75,000–$100,000		4(6)	4(6)	
> $100,000		5(13)	5(8)	

^1^lifetime use of BBT;^2 ^mean age ± standard deviation (range);

^3^Asian/Pacific Islander/Indian

Overall, education level significantly influenced the use of BBT, p = 0.0006. Among users, more patients had college degrees (28 [30%]) as compared with nonusers, (12 [12%], p = 0.003). In contrast, 42 (40%) nonusers finished high school versus 23 (24%) of users, p = 0.023. There were no significant differences between users and nonusers in other demographic variables.

### Cardiovascular diseases, other medical conditions and medications used

The distribution of cardiovascular and noncardiovascular diseases reported by the sample is presented in Table 2. The mean number of disease diagnoses carried by users and nonusers were 4 (range 1 – 10) and 3 (range 1 – 8), respectively, p = 0.723, while the mean number of cardiovascular diagnoses for users versus nonusers were 3 (range 1 – 6) and 3 (range 1 – 5), respectively, p = 0.134. The most common cardiovascular conditions diagnosed in users and nonusers were hypertension, 56 (24%) and 65 (27%), followed closely by coronary heart disease, 45 (19%) and 54 (18%), respectively. Distribution of other cardiovascular diseases between users and nonusers was similar as well. Similarly, the mean number of noncardiovascular diagnoses reported by users and nonusers of BBT were not significantly different: 2 (range 1 – 5) and 1 (range 1 – 5), respectively, p = 0.288.

**Table 2:** Distribution of Cardiovascular and Noncardiovascular Diseases

	**Users**	**Nonusers**	**p value**
**Average number of diseases**	4	3	NSS
**Range**	1 – 10	1 – 8	
**Average number of cardiovascular diseases**	3	3	NSS
Arrhythmia	25	32	
Congestive Heart Failure	38	52	
Coronary Heart Disease	45	54	
Hyperlipidemia	31	43	
Hypertension	56	65	
Other^1^	12	14	
Post-heart Transplant Recipient	1	2	
Thromboembolic Disease	13	16	
Valvular Heart Disease	11	14	
**Average number of noncardiovascular diseases**	2	1	NSS
Arthritis	25	17	
Cancer	3	4	
Diabetes Mellitis	33	33	
Gastrointestinal Disease	19	13	
Hypothyroidism	5	11	
Nervous System Disorder	8	9	
Ocular Disease	6	6	
Other^2^	30	13	
Pulmonary Disease	12	13	
Renal Disease	7	12	

^1^aneurysm, peripheral vascular disease, infective endocarditis, pulmonary hypertension, congenital heart disease;

^2^allergic rhinitis, scleroderma, systemic lupus erythematosis, Raynaud's disease, benign prostatic disease, osteoporosis

The mean number of traditional prescription and nonprescription medications taken by the users of BBT (7 [range 1–15]) and nonusers (7 [range 0–18]) was not significantly different, p = 0.445. The most common cardiovascular medications prescribed for both users and nonusers were diuretics (71%, 73%), followed by aspirin (56%, 43%) and beta-blockers (49%, 50%). The cardiovascular and noncardiovascular medications taken by both groups were not different.

### Types and patterns of BBT use

The BBT products utilized by cardiovascular patients are presented in Table 3. The mean number of BBT products utilized by cardiovascular patients was two. Vitamin E (41, [44%]) was the most commonly utilized BBT, followed by vitamin C, (30 [32%]). The prevalence of the remaining BBT is summarized in Table 3.

**Table 3:** All Biological Based Therapies Utilized by Cardiovascular Patients (at any time)

**Product**	**Number**	**Reasons for Use (per patient)**
AA#5 (anti-arthritis)	1	treat arthritis
Acidophillus	1	health
Aloe vera	4	headache, stomach gas, skin pigmentation
Atomic Drops	1	treat headache
Bee Pollen	1	prevent cold
Beta carotene	5	maintain good health, energy, improve heart contraction
Bioflavinoid	1	bone health instructed by chiropractor
Calcium	19	supplement, prevent osteoporosis, improve heart function
Chamomile	2	stomach ache, improve heart condition
Chromium picolante	2	supplement for heart condition, muscle strength
Coenzyme Q 10	11	improve heart contraction, supplement to diet
Dexatrim	1	weight lost
DHEA^1^	1	supplement
Echinacea	5	prevent or treat cold, flu, stay healthy, boost immune system
Fish Oil	12	decrease cholesterol, maintain circulation and good health, scleroderma
Folic Acid	3	supplement, help with heart condition, sickle cell anemia
Garlic	5	decrease cholesterol, help maintain good health
Ginkgo biloba	5	antioxidant, enhance memory, energy
Ginseng	4	increase energy, stamina, virility
Glucosamine/chondroitin	10	treat arthritis, decrease joint pain
Golden seal	1	body cleaner
Grapeseed oil	1	preserve health
Green tea	3	decrease cholesterol, improve circulation
Mixed herbal tea	1	Sooth stomach upset, anxiety
Insulin leaf tea	1	diabetes
Iron supplement	4	anemia, increase energy
Lecithin	2	improve heart condition and circulation, decrease cholesterol
Alpha-Linolenic acid	1	improve heart condition
Magnesium	8	supplement, antioxidant, improve metabolism, improve heart function
Multivitamins	24	supplement, energy, sickle cell anemia
Primrose oil	1	scleroderma
Saint John's Wart	1	depression
Saw palmetto	1	for prostate health
Selenium	4	antioxidant, supplement, improve heart condition
Strong bark	1	help with heart condition
Valerian	4	decrease anxiety, improve sleep, to decrease blood pressure
Vitamin B complex	17	supplement, improve heart condition, decrease leg cramps, energy
Vitamin B12	2	supplement, anemia
Vitamin C	30	antioxidant, supplement, help with heart condition, improve circulation, strengthen immune system, prevent or treat cold
Vitamin E	41	antioxidant, supplement, help with heart condition, increase energy, decrease cholesterol, improve circulation, thin blood, treat hypertension
Yohimbine	1	increase energy, stamina, virility
Zinc	4	supplement

^1^dehydroepiandrosterone

The patients reported that their physicians were aware of their BBT use in 60% of the instances and pharmacists were aware in 32% of the cases. Only 33% of users reported that they were asked about BBT use during a history/physical examination by a health care professional. The patients were not surveyed about their pharmacists' assessments of BBT use.

### Perceived benefits and attitudes towards BBT

A greater percentage of users (74.5%) of BBT reported that they believed these products to be safe substances as compared with nonusers (26.2%), p < 0.001. Likewise, significantly more users believed that BBT was effective (70.2%) compared to nonusers (30.1%), p < 0.001. More nonusers (72%) than users (45%) did not know whether BBT products work better, as well as, or worse than traditional medications, p < 0.001. More users than nonusers believed that BBT works as well as traditional medications (30.9% versus 9.7%, p < 0.001). Concerning adverse effects, more users (44.7%) of BBT than nonusers (17.5%) believed that BBT causes fewer side effects than traditional medications, p < 0.001. At the same time, 32.2% of users and 62.1% of nonusers did not know whether BBT causes more or fewer side effects than traditional prescription medications, p < 0.001.

### Potential drug-BBT interactions

Examination of patients' prescription and nonprescription medication profiles and BBT utilized, revealed 42 potential drug-BBT interactions. The onset of the interaction and the degree of severity were classified according to that used by the MicroMedex HealthCare Series Integraded Index [28] and the published literature [29-43]. Suspected or potential interactions were communicated to the patients and their primary physicians. The most common interaction identified was coadministration of aspirin and vitamin E (16 cases) that could potentially result in an increased risk of bleeding due to additive inhibition of platelet aggregation [29-31]. Similar effects may result from coadministration of clopidogrel and vitamin E; this potential interaction was recognized in five cases [31]. Five instances of potential interaction between warfarin and vitamin E were identified and close monitoring of the International Normalized Ratio (INR) was recommended [32-34]. A list of other potential drug-BBT interactions that were identified is presented in Table 4.

**Table 4:** Potential Drug-Biological Based Therapies Interactions & Management

**Medication**	**Alternative Pharmacotherapy Product**	**Potential Interaction and Management**	**Number of Patients**	Onset^28^*	**Level of Severity^28^**
Warfarin	Green tea	Green tea may antagonize warfarin effects. Monitor INR.	1	Delayed	Moderate
Warfarin	Coenzyme Q 10	Coenzyme Q 10 may have procoagulant effects and may decrease response to warfarin. Monitor INR.	2	Delayed	Moderate
Warfarin	Vitamin E	Vitamin E may potentiate warfarin effects. Monitor INR and signs and symptoms of bleeding.	5	Delayed	Moderate
Warfarin	Garlic	Garlic has antiplatelet effects, and risk of bleeding may be increased. Monitor INR and signs and symptoms of bleeding.	1	Delayed	Major
Warfarin	Ginkgo biloba	Ginkgo inhibits platelet aggregation, risk of bleeding may be increased. Monitor INR and signs and symptoms of bleeding	2	Delayed	Major
Warfarin	Fish oils	Concomitant use of warfarin and fish oils may increase risk of bleeding. Monitor INR and signs and symptoms of bleeding.	1	Not specified	Not specified
Aspirin	Vitamin E	Coadministration of vitamin E and aspirin may increase the risk of bleeding. Monitor for signs and symptoms of bleeding	16	Not Specified	Not specified
Aspirin	Garlic	Additive antiplatelet effects may occur with coadministration of garlic and aspirin, risk of bleeding may be increased. Monitor for signs and symptoms of bleeding	1	Delayed	Moderate
Aspirin	Fish oils	Concomitant use of aspirin and fish oils may increase risk of bleeding. Monitor for signs and symptoms of bleeding	3	Not specified	Not specified
Aspirin	Ginko biloba	Ginkgo inhibits platelet aggregation; risk of bleeding may be increased. Monitor for signs and symptoms of bleeding.	1	Delayed	Major
Clopidogrel	Vitamin E	Concomitant use of vitamin E and clopidogrel may increase the risk of bleeding. Monitor for signs and symptoms of bleeding	5	Not specified	Not specified
Clopidogrel	Garlic	Additive antiplatelet effects may occur, risk of bleeding may be increased. Monitor for signs and symptoms of bleeding	1	Delayed	Major
Clopidogrel	Coenzyme Q	Coenzyme Q 10 may have procoagulant effects, may partially antagonize antiplatelet effect of clopidogrel.	1	Not specified	Not specified
Insulin	Insulin leaf	Enhanced hypoglycemic effects. Patient was advised to discontinue insulin leaf while receiving insulin for glycemic control.	1	Delayed	Moderate
Paroxetine	Saint John's Wort	Saint John's Wort induces cytochrome P450 3A4 enzyme and has mechanism of action similar to serotonin reuptake inhibitors. Administering it concomitantly with paroxetine will enhance the toxicity of paroxetine. Patient was advised to discontinue the use of Saint John's Wort	1	Rapid	Severe

INR = International Normalized Ratio

* Delayed onset of interactions occurs after multiple doses of both agents.

## Discussion

Despite a wide array of available BBT for cardiovascular conditions, studies evaluating the prevalence of their usage are limited. A review of the literature at the time of study initiation (January 2001) and more recently (June 2004) identified five studies in this area, and only a few included a broad spectrum of cardiovascular disease patients [21-25].

Wood et al. conducted a telephone survey in 107 patients randomly selected from the "Improving Cardiovascular Outcomes in Nova Scotia" database [24]. A majority of patients (64%) in this study utilized CAM. Ackman and colleagues evaluated the patterns of BBT use by patients with congestive heart failure (CHF) [21]. Out of 180 CHF patients, 59% used vitamins and minerals and 38% used herbal or health food products. Liu and associates evaluated the prevalence of CAM used in 263 patients undergoing cardiovascular surgery [22]. Seventy-five percent of respondents utilized CAM including vitamins (53.6%), nutritional therapy (17.1%) and herbs (9.9%). Compared with non-CAM users, users were older (p = 0.027), belonged to the Caucasian racial group, (p = 0.001) and had a higher level of education (p = 0.017). Additionally, the evaluation of attitudes towards the effectiveness of CAM revealed that users were more likely to believe that CAM would work in a complimentary manner with conventional medical treatments, p < 0.05. Furthermore, more users than nonusers believed that CAM would promote general health and wellness, p < 0.05. Of the patients surveyed, only 17% reported discussing CAM with their medical doctors. Another recent study evaluated the use of CAM among 246 patients attending a cardiac clinic prior to cardiac surgery [23]. A total of 182 (80.9%) patients used CAM, and 12.9% utilized megavitamins. Another study, conducted in Canada, focused on the use of over-the-counter medications and herbal products among patients with cardiac diseases, the majority of whom were diagnosed with coronary artery disease (74%) [25]. The authors reported that 23% of the patients used multivitamin or multivitamin/mineral products. Overall, the results of these five studies confirm a high prevalence of all kinds of CAM used, including BBT among patients with cardiovascular diseases.

### Biological based therapy used by cardiovascular patients

In our study, the lifetime prevalence of BBT use in the sample of 198 cardiovascular patients was 47.5%, which is very similar to the results reported by Eisenberg and colleagues in the general population (42%) [3]. Comparisons with other investigations of alternative medicine use in cardiovascular patients are difficult to make due to the various definitions of "alternative medicine" [21-25]. Some studies included items such as alternative procedures including acupuncture, while others included "pharmacotherapy" only (herbs, BBT, CAM and nutritional supplements). In addition, certain studies looked at only one cardiovascular disease while others looked at a broad range of patients with multiple diseases. Furthermore, different studies also used different measurements of incidence and prevalence; i.e., some investigators focused on lifetime use, others reviewed the previous 12-month history only, yet others limited their findings to BBT use in the 14 days prior to the investigation. Regardless, results indicated that a high percentage of patients with cardiovascular diseases are taking some kind of BBT (almost one out of every two patients). Health care professionals need to be aware of these findings and routinely inquire about BBT use by patients when taking a medication history.

Similar to Ackman's study [21], the most frequently utilized BBT in the present study of cardiovascular patients was vitamin E, (41 [43.6%]). The popularity of vitamin E among cardiovascular patients is not surprising due to the alleged benefits of this vitamin in heart disease from early literature [12-14], and its relative availability in pharmacies, health food stores and supermarkets. As was the case with vitamin E, the use of vitamin C in the current investigation, (30 [31.9%]) was similar to previously reported results, being the second most common product utilized by cardiovascular patients [21].

Similar to other studies [1,3,6,21], the current study confirmed that based on the patients' reports, a high percentage of physicians (40%) and pharmacists (68%) were not aware of BBT used by cardiovascular patients. Use of BBT by the patients in this study was not routinely discussed with health care providers during medical history evaluation (33%). This is an alarming but not an isolated finding. On average, cardiovascular patients consumed 7 (range, 1 – 18) prescribed medications, and 2 (range, 1 – 12) BBT products. Considering the complex medical treatment received by cardiovascular patients, addition of unmonitored BBT to the patients' regimens may place the users of these therapies at a greater risk for the development of adverse events and interactions with prescribed medications. As addressed by this analysis, 42 potential drug-BBT interactions were identified.

### Demographics impact on biological based therapy use in cardiovascular patients

Among the demographic variables collected in this analysis, no parameter other than the level of education had a significant impact on whether patients used BBT. The present investigation revealed that the education level among users (63% received some college or college and graduate school education) of BBT was higher than that of the nonusers (32%), p < 0.001. This finding has also been reported in other studies [3,6]. Higher level of education has been shown to significantly influence the use of alternative products and services [3]. Education level is sometimes directly related to economic status, thus the patients may have more resources to spend on BBT. Similarly, better-educated consumers may be more likely to be exposed to various less conventional forms of healthcare reading about their illnesses and treatment options. Educated patients also might be less inclined to accept their physicians' knowledge and expertise, and may seek other treatment options.

### Perceived safety and efficacy of BBT

The majority of BBT users (as compared to nonusers) in the current investigation believed that BBT products are safe, effective and cause fewer side effects as compared to prescription medications. These results are consistent with the findings by other investigators [6,21], supporting a logical conclusion that patients who believe that BBT are safe and effective, are more likely to use them.

### Study limitations

Several limitations of the study need to be addressed. Those that are intrinsically related to survey data collection in general include potential bias of responders and nonresponders. Since participation in this study was voluntary, patients who chose to participate in the study may be more motivated and knowledgeable about the subject of BBT as compared to those who refused to participate. Therefore, the results of this study may not be applicable to the entire cardiovascular patient population. Also, a cross-sectional nature of this study precludes drawing any definitive conclusions regarding cause and effect relationships. For example, one cannot definitively conclude that more education will significantly effect one's decision to take BBT because other factors such as exposure to these types of products may have influenced more educated consumers to take these products.

As with other surveys, recall bias cannot be eliminated as data collection relied on patients' self-report, rather than objectively documenting BBT use. Additionally, with surveys there is always a possibility of misunderstanding the questions and responses, miscommunication between the interviewer and the patients, and inaccurate recording of the information. The majority of the survey collected factual information (i.e., demographics and BBT usage history). However, the questions that evaluated patients' perception on the safety and efficacy of conventional medicine and BBT were not validated.

This study was conducted in a large urban inner city hospital; the results may not be extrapolated to other cities or clinical settings. Another limitation may be the exclusion of non-English speaking patients, which may be particularly important since there are certain ethnic groups who are documented to use more BBT than are other ethnic groups (e.g., persons of or from Chinese descent utilize traditional Chinese herbal medications). Finally, terminology varies greatly in the published literature. Some authors use the term CAM in all instances, others differentiate CAM from BBT, etc. As a result, it is very difficult to compare published reports with certainty.

## Conclusion

In recent years, the interest of using BBT in disease management has increased dramatically in the medical and layman communities. The amount of valid scientific research in this area of therapy continues to increase. Yet, there are still many unknowns concerning BBT, especially in the area of adverse effects and drug interactions. The finding of a high prevalence of BBT (47.5%) use among cardiovascular patients and the lack of communication between patients and their physicians/pharmacists should be addressed by the health care community. Higher education level, as shown in the present study and other previous investigations [1,3,6,22], is associated with an increased use of BBT, but it does not necessarily mean that these patients are aware of the potential detrimental effects of BBT, as demonstrated in the current study. In cardiovascular patients, the perceived effectiveness and safety of BBT, and assumed lack of side effects of these products as opposed to traditional medications, highlights an area for further education. A high incidence of potential drug-BBT interactions was also identified in this study (42 interactions in 94 users). Given that the use of BBT can have a direct effect on patient care, and users of these therapies do not always voluntarily report their use of these products to their providers, health care professionals need to inquire about BBT use routinely. Collecting complete patient histories and educating patients about potential dangers and possibilities of adverse effects and interactions between prescription medications and BBT (or other CAM) will lead to better overall patient care.

## Competing interests

The author(s) declare that they have no competing interests.

## Authors' contributions

LC participated in literature review, protocol development, collected and analyzed data, and submitted the manuscript for publication. DB collected the data. JWMC participated in study design, data analysis and preparation of manuscript. VR participated in design development and data analysis. HLK proposed the project and reviewed the manuscript. GCC, JM and BM participated in protocol implementation and manuscript review. All authors read and approved the manuscript.

## Pre-publication history

The pre-publication history for this paper can be accessed here:



## Supplementary Material

Additional File 1 Appendix A – Biological based therapy survey, It's a survey tool utilized in the study to collect patient data. Click here for file
