# Supplementary material for: Use of biological based therapy in patients with cardiovascular diseases in a university-hospital in New York City
Source: BMC Complement Altern Med. 2005 Mar 3;5:4. doi: 10.1186/1472-6882-5-4 (PMC555537; doi:10.1186/1472-6882-5-4)
Supplement: Additional File 1 — Appendix A – Biological based therapy survey, It's a survey tool utilized in the study to collect patient data. [file 1472-6882-5-4-S1.doc]

# Additional file 1- Appendix A, Biological based therapy survey

**Patient Study ID**: □ □ □

**Patient Hospital ID#** ______________________________________________

1. **Age of patient**

______18 – 29 ______30 – 39 _______40 – 49

______50 – 59 ______60 – 69 _______70 – 79

______80 – 89 ______ 90 - 100

1. **Gender**

______Male ______Female

1. **Where were you born? If not in the U.S., indicate what country are you from?** ____________________________________________________________________
2. **If not born in the U.S., when did you come to the U.S.?**______________________
3. **Native language**_______________________________________________________
4. **What race or ethnic group would you consider yourself?**

______White, not Hispanic ______Black, not Hispanic

______Hispanic ______Asian/Pacific Islander

______Alaskan Native or Native American ______Other, specify

**7. What is your marital status?**

______Single ______Married

______Widowed ______Divorced/separated

**8. What level of education did you complete?**

______< High school _____High school

______Some college _____College graduate

______Graduate degree ______Other, specify

**9. What is your yearly household income?**

______< $10,000 ______$10,000 – $30,000

______$30,000- $50,000 ______$50,000 – $75,000

______$75,000 – $100,000 ______> $100,000

**10. What is your current working status?**

______Full – time _______Part-time _______Retired

______Unemployed _______Self-employed

**11. How do you pay for your medical care?**

______Cash ______Medicare/Medicaid

______HMO ______Other, specify

**12. Allergies:**_________________________________________________________

**13. What type(s) of cardiovascular disease(s) do you have, for how long, and how long have you been treated for it (them)?**

| **√** | **Cardiovascular Disease(s)** | **When diagnosed (yr)** | **Duration of Treatment (yrs)** |
| --- | --- | --- | --- |
|  | Congestive heart failure (heart does not pump as well as it should) |  |  |
|  | Hypertension (high blood pressure) |  |  |
|  | Coronary heart disease (hardening of the arteries of the heart, chest pain, heart attack) |  |  |
|  | Thromboembolic disease (blood clotting disease) |  |  |
|  | Valvular heart disease (disease of the valves of the heart) |  |  |
|  | Post-heart transplantation (after transplantation of the heart) |  |  |
|  | Other, specify:_____________________________ _________________________________________  _________________________________________ |  |  |

**14. Other than the cardiovascular disease(s), what other medical conditions are**

**you suffering from and for how long?**

| **Number** | Medical Condition | When diagnosed (yr)? |
| --- | --- | --- |
| **1.** |  |  |
| **2.** |  |  |
| **3.** |  |  |
| **4.** |  |  |
| **5.** |  |  |
| **6.** |  |  |
| **7.** |  |  |

**15. Please, tell me about all of the medications (prescription and over-the-**

**counter) you were taking prior to the current admission?**

*Prescription Medications:*

| **Number** | **Prescription Medication** | **Dose, Route and Frequency** |
| --- | --- | --- |
| **1.** |  |  |
| **2.** |  |  |
| **3.** |  |  |
| **4.** |  |  |
| **5.** |  |  |
| **6.** |  |  |
| **7.** |  |  |
| **8.** |  |  |

*Over-the-counter or Nonprescription Medications:*

| **Number** | **Over-the-counter Medication** | **Dose, Route and Frequency** |
| --- | --- | --- |
| **1.** |  |  |
| **2.** |  |  |
| **3.** |  |  |
| **4.** |  |  |
| **5.** |  |  |
| **6.** |  |  |
| **7.** |  |  |
| **8.** |  |  |

**16. How would you rate your satisfaction with your current medications for cardiovascular disease(s)?**

______Very satisfied

______Satisfied

______Neither satisfied nor unsatisfied

______Unsatisfied

______Very unsatisfied

17. Have you ever experienced an adverse reaction from a medication?

______Yes ______No

If yes, specify:______________________________________________________

__________________________________________________________________

**18. Have you ever used an herbal or dietary supplement? (if no, go to**

**question 24, 25, 28, and 29)**

______Yes ______No

1. Have you used any of the herbal or dietary supplements in the past 12 months?

______Yes ______No

20. If you used herbal or dietary supplement(s), describe your pattern of using dietary supplements?

______Once ______Occasionally

______Regularly ______All the time

1. Within the last 12 months, name the herbal and/or dietary supplement(s)

that you have used, the way you took these products, and reasons for using these products?

| Number | Dietary Supplement | Dose, Route and Frequency | Reasons for Use |
| --- | --- | --- | --- |
| 1. |  |  |  |
| 2. |  |  |  |
| 3. |  |  |  |
| 4. |  |  |  |
| 5. |  |  |  |
| 6. |  |  |  |
| 7. |  |  |  |
| 8. |  |  |  |
| 9. |  |  |  |
| 10. |  |  |  |

**22. How did you learn about herbal/dietary supplement(s)?**

______Health care provider, specify ______Advice from friend/relative

______Magazine/newspaper ______Internet

______Herbalist or nonmedical provider ______Other, specify

**23. Where do you buy your herbal/dietary supplement(s)?**

______Supermarkets ________Pharmacies

______Health food stores ________Alternative medicine magazines

______Internet ________Alternative medicine provider

______Other, specify

24. Do you believe herbal/dietary supplements are safe?

_______Strongly agree _______Agree

_______Neutral _______Disagree

_______Strongly disagree

25. Do you believe herbal/dietary supplements are effective?

_______Strongly agree _______Agree

_______Neutral _______Disagree

_______Strongly disagree

1. What are some of the potential side effect(s) of each of the dietary

supplement you take?

________________________________________________________________________________________________________________________________________________________________________________________________________________________________________________________________________________________________________________________________________________________

1. Name other drugs or foods that you may need to avoid while taking your

dietary supplement(s)

__________________________________________________________________

__________________________________________________________________________________________________________________________________________________________________________________________________________________________________________________________________

1. How do you think dietary supplements work in comparison to traditional

medications?

______Better than traditional medication

______As good as traditional medication

______Worse than traditional medication

______Do not know

1. Do you believe herbal/dietary supplements cause more or less side effects

than traditional medications?

_____More _____Less _____About the same _____Do not know

30. How did you pay for your dietary supplements?

­­­­­_____Insurance _____Cash _____Other, specify

# 31. Have you ever experienced an adverse reactions from an herbal/dietary

# supplement(s)?

______Yes _______No

If yes, specify:______________________________________________________

**32. Is your physician aware of your use of herbal/dietary supplements?**

_______Yes _______No

If no, why?________________________________________________________

**33. Is your pharmacist aware of your use of herbal/dietary supplements?**

_______Yes _______No

If no, why?________________________________________________________

# 34. Do your health care providers, meaning physicians, pharmacists, nurses ask you about your use of herbal/dietary supplement(s)?

______Yes _______No

If yes, which ones__________________________________________________

35. Approximately, how much money did you spend on herbal/dietary

supplement(s) in the last year?

_____< $50 _____$50 – 100

_____$100 – 150 _____$150 – 200

_____> $200

36. Would you recommend use of herbal/dietary supplement to another patient with similar medical condition(s) to yours?

_____Yes _____No

If yes, have you?____________________________________________________
